# Resumption of attention-deficit hyperactivity disorder medication in early adulthood: findings from a UK primary care prescribing study

**DOI:** 10.1007/s00787-019-01325-5

**Published:** 2019-04-04

**Authors:** Tamsin Newlove-Delgado, Tamsin J. Ford, Willie Hamilton, Astrid Janssens, Ken Stein, Obioha C. Ukoumunne

**Affiliations:** 1 University of Exeter Medical School, St Luke’s Campus, Heavitree Road, Exeter, EX1 2LU UK; 2 Child and Adolescent Psychiatry, University of Exeter Medical School, Exeter, UK; 3 Primary Care Diagnostics, University of Exeter Medical School, Exeter, UK; 4 Department of Public Health, University of Southern Denmark, Odense, Denmark; 5 Public Health, NIHR CLAHRC South West Peninsula (PenCLAHRC), University of Exeter Medical School, Exeter, UK; 6 Medical Statistics, NIHR CLAHRC South West Peninsula (PenCLAHRC), University of Exeter Medical School, Exeter, UK

**Keywords:** ADHD, Transition, Prescribing, Adolescents

## Abstract

This study aimed to examine the resumption of attention-deficit hyperactivity disorder (ADHD) prescriptions in early adulthood in young people whose ADHD prescriptions stopped in adolescence. Whilst prescribing studies indicate that the proportion of those with ADHD stopping treatment in late adolescence remains in excess of the proportion expected to be symptom free, very few studies have examined patterns of resumption amongst young adults previously prescribed medication. Primary care records from the UK Clinical Practice Research Datalink from 2008 to 2013 were used to examine the outcome of resumption of ADHD prescriptions from age 20 years in a sample of cases with ADHD whose prescriptions stopped aged 14–18 years. A Cox regression model was fitted to explore variables that could theoretically be associated with resumption of prescriptions. Of 1440 cases, 109 (7.6%) had their ADHD prescriptions resumed. Characteristics associated with an increased probability of resumption included female gender, learning disability, referral to adult mental health services, and prescription of antipsychotic medication. In this study, only a small proportion of adolescents who stopped ADHD medication subsequently resumed their prescriptions in primary care. Those that did resume were a more complex group. As many vulnerable individuals with ongoing ADHD symptoms may not have the resources required to surmount the barriers to re-enter services, the implication is that not all those who could benefit from resuming medication are able to do so. The findings raise questions around whether current care models are flexible enough and whether primary care services are adequately supported in managing this group.

## Introduction

Attention-deficit hyperactivity disorder (ADHD) is now accepted to be a condition which can persist throughout life. Follow-up studies suggest that up to three-quarters of young people diagnosed as children continue to experience symptoms into adulthood, even if they no longer meet full diagnostic criteria [1]. These symptoms, which include inattention, impulsivity, and hyperactivity, can undermine the fulfilment of adult responsibilities such as further education, employment, intimate relationships and parenting, and are associated with risk-taking behaviours, accidents and injuries [2–5].

Consequently, much recent research has focussed on ADHD management during the transition to adulthood, and in particular on the prescription of ADHD medication. In studies from the UK, USA and Europe [6–9], the proportion of young people stopping medication during this period appears to be in excess of the proportion expected to be symptom free from follow-up studies [1]. For those with ongoing symptoms, cessation of medication is likely to represent a complex combination of choice and circumstance. During adolescence, young people are expected to become involved in decision making about their treatment and may experiment with starting and stopping medication and with alternative coping strategies [10–12]. Medication is also often perceived as an intervention intended to allow the young person to cope with school and once examinations are finished, the adverse effects may be seen to outweigh the benefits [12–14]. External factors may influence ongoing prescribing, such as a lack of services or expertise to oversee medication management in adults, or a lack of support, resource and preparation for transition to adult services that leads both services and young people to disengage even before the age boundary for the service is reached [14–16].

Stopping medication can have positive outcomes for many young people, but for others, cessation may be detrimental and destabilising at a vulnerable life stage. Arguably, we would therefore expect to see a prescribing pattern whereby some adolescents with continuing symptoms stop medication but later seek to restart treatment as young adults. Guidance from the UK National Institute for Health and Care Excellence (NICE) now recognises that people with ADHD previously treated as children may return to services after the age of 18 years, seeking support and treatment for continuing ADHD symptoms [17].

This group of young adults who do not undergo transition into an adult service for their ADHD but then seek to re-enter services and potentially resume treatment have been little studied to date. Therefore, there is a gap in knowledge about the scale and needs of this group, which is important for planning services and training. Very few studies have examined the patterns of resumption amongst young people previously prescribed medication. Notably, whilst the USA-based large multimodal treatment of attention-deficit hyperactivity disorder (MTA) trial reported on ‘stopping and restarting’ patterns in adolescents, it has not published data on resumption after the age of 18 years [12]. In the UK, Wong et al. described resumption in the General Practice Research Datalink from 1999 to 2006 amongst patients who had stopped after the age of 15 years [18]. Only 56 of the 407 individuals who stopped restarted treatment during the follow-up period, the majority in the first year after cessation; however, this study took place prior to the publication of the first UK NICE ADHD guidance that referred to adults in 2008 [19].

We aimed to explore resumption of ADHD medication prescriptions in early adulthood (from age 20 onwards) amongst young people whose prescriptions stopped in adolescence (aged 14–18 years) using primary care prescribing data from the UK Clinical Practice Research Datalink from 2005 to 2013. The age range of 14–18 years for cessation was chosen as it corresponds to adolescence and the period of preparation for transition to adult health-care services; this is the time at which young people are most likely to cease their ADHD medication [8, 9]. We studied resumption from the age of 20 onwards, to capture prescribing in adult services rather than extended prescribing in child services.

Our chief research questions were:

- What proportion of young people who have previously been prescribed ADHD medication and stopped resume prescriptions in early adulthood, and what is the probability of resuming medication from age 20 years onwards?
- What background characteristics are associated with resumption?

## Methods

### Study design

This study was a secondary analysis of data from the Clinical Practice Research Datalink (CPRD), a large UK clinical database run by the Medicines and Healthcare Products Regulatory Agency (MHRA). The primary care section of the database contains the records of over 11 million patients and is contributed to by more than 670 GP practices across the UK, covering over 6% of the population [20]. CPRD uses software to extract anonymised data from participating practices’ IT systems. Records of each patient are coded by GPs and practice staff according to NHS coding schemes, and all primary care-issued prescriptions are automatically captured. All protocols using patient-level data from the CPRD are reviewed and approved by the Independent Scientific Advisory Committee on behalf of the National Research Ethics Service Committee. This study protocol (13_213) was granted approval in 2013.

### Patients

The sample for this analysis used a subset of a larger dataset of patients (*N* = 9390) with a recorded diagnosis of ADHD using NHS coding schemes (described in Newlove-Delgado et al. [9]). Recorded psychiatric diagnoses were identified using CPRD medical codes and READ term codes used in UK primary care, which map to ICD-10 categories [21]. Psychotropic prescribing (including for ADHD medication) was categorised using CPRD product codes referring to British National Formulary categories [22]. The dataset included patients aged between 10 and 20 years in 2005, and the study period ran from 1 January 2005 until 31 December 2013.

To be included in this analysis, patients had to:

- Have at least 1 year’s worth of prescribing records for an ADHD medication, to ensure this was a regular prescription and not a ‘trial’ of medication.
- Have their last recorded prescription for an ADHD medication issued between the ages of 14 and 18 years (inclusive); with no further ADHD prescribing recorded until the age of 20 years if at all.
- Have at least 1 year’s worth of follow-up data in the CPRD from the age of 20 years.

### Analysis

Resumption was defined as having at least one recorded prescription of ADHD medication at the age of 20 years or over. We reported the percentage of included cases who resumed prescriptions and the age of restarting, and the percentage of those who resumed prescriptions by age at stopping. The percentage of those who resumed medication was then reported by key characteristics (gender, non-ADHD prescription status, recorded referral to adult mental health services and non-ADHD psychiatric diagnoses). We also reported the prescription of non-ADHD psychotropic medication in individuals who did and did not resume ADHD prescriptions and used the Chi squared test to compare the proportions.

We then performed survival analysis to examine the time to resumption of ADHD prescriptions. The observation period for the survival analysis began from the year of the case’s 20th birthday and continued until a new prescription for an ADHD medication was recorded (resumption), until records for the patient were no longer available or the study period ended (31 December 2013)—i.e., they were censored. The median time to resumption was reported and the distribution of time to resumption was summarised using the Kaplan–Meier estimator. A Cox regression model was fitted to explore variables that could theoretically be associated with resumption of prescription, based on the previous descriptive analysis. These included gender, referral to adult psychiatry, non-ADHD psychotropic primary care prescribing aged 18 and under or aged 19 and over, and recorded psychiatric comorbidities, as well as age at stopping medication. Each variable was initially examined in an unadjusted Cox regression model, reporting hazard ratios. Predictor variables with a *p* value less than 0.05 were then included in a multivariable (adjusted) Cox regression model, with the same tests of the proportional hazards assumption applied. The proportional hazards assumption was checked by examining Schoenfeld residuals and plotting Nelson–Aalen cumulative hazard estimates. Statistical analyses were carried out using Stata software (version 14) [23].

### Findings

There were 1440 young people in the analysis who met our inclusion criteria. Of these, 1293 were male (89.8%), 395 (27.4%) had a prescription for a non-ADHD psychotropic medication at any point, 348 (24.2%) had a recorded diagnosis of another psychiatric disorder (excluding learning disability), and 64 (4.4%) had a recorded learning disability.

In our sample, 109 participants (7.6%, 95% CI 6.3–9.1%) had their prescriptions resumed at the age of 20 years or over. Of the 147 females in the sample, 17 (11.6%) resumed medication, compared to 92 of 1293 males (7.1%).

#### Ages of stopping and resuming medication

Of the 1440 participants, 427 (29.7%) stopped medication at age 16 years, which was the most common age for cessation, although 372 participants (25.8%) stopped medication at 17 years and 319 (22.2%) at 18 years.

Table 1 displays the percentage of young people in our sample whose prescriptions were resumed at specific ages. The majority of those who did restart did so before the age of 22 years.

**Table 1:** Outcomes of young people who stopped ADHD prescriptions aged 14–18 years

Outcome	*n*	Percentage
Did not restart	1331	92.4
Restarted age 20	66	4.6
Restarted age 21	19	1.3
Restarted age 22	15	1.0
Restarted age 23	6	0.4
Restarted age 24	2	0.1
Restarted age 25	1	0.1
Total	1440	100

Once prescriptions were resumed, the median duration of persistence was 1 year (interquartile range 1–4 years) with the range from less than a year to 5 years; cases still prescribed medication at 6 years or more post-resumption were censored (see “Methods”).

Overall, the later the young person stopped medication, the more likely they were to restart prescriptions in early adulthood. Only 6 (5.3%) of the 113 who stopped medication at age 14 years resumed medication, compared to 42 (13.2%) of the 319 who stopped medication aged 18 years (Table 2).

**Table 2:** Resumption status by age of stopping ADHD prescription

Age at stopping prescription	Number restarting prescription (%)
Age 14 (*N* = 113)	6 (5.3%)
Age 15 (*N* = 209)	5 (2.4%)
Age 16 (*N* = 427)	24 (5.6%)
Age 17 (*N* = 372)	32 (8.6%)
Age 18 (*N* = 319)	42 (13.2%)

### Resumption of primary care prescriptions for ADHD medication by characteristics

Table 3 describes the number and percentage of young people with different characteristics, whose ADHD prescriptions resumed in early adulthood. Resumption levels were highest amongst cases with a recorded referral to adult mental health services, those with a learning disability, and those prescribed a non-ADHD psychotropic medication (Table 3). In terms of psychiatric diagnoses, of the 35 cases in the sample with a substance misuse diagnosis, 6 resumed medication for their ADHD, compared to 9 of the 92 with conduct or oppositional defiant disorder.

**Table 3:** Resumption of primary care prescriptions for ADHD medication by recorded characteristic

Characteristic	Number resuming medication (%)
Prescription for non-ADHD psychotropic (*N* = 395)	52 (13.2%)
Referral recorded to adult mental health services (*N* = 142)	28 (19.7%)
Any non-ADHD psychiatric disorder (excluding learning disability) (*N* = 348)	36 (10.3%)
Conduct or oppositional defiant disorder (*N* = 92)	9 (9.8%)
Learning disability (*N* = 64)	12 (18.8%)
Autism spectrum disorder (*N* = 112)	13 (11.6%)
Anxiety or depression (*n* = 125)	15 (12.0%)
Substance misuse (*N* = 35)	6 (17.1%)
All cases (*N* = 1440)	109 (7.6%)

### Time to resumption

The cumulative percentage of prescription resumption rose with age, but then remained stable from the age of 24 years; additionally, far fewer cases remained in the survival analysis after this point with a high proportion being censored. The estimated cumulative probability of resumption at 1 year (by age 20) was 4.6% (95% CI 3.5–5.8%); at 3 years (by the age of 22), this had risen to 7.6% (95% CI 6.3–9.2%) and at 5 years (by age 24) this was 9.7% (95% CI 7.7–12.0%).

### Factors associated with resumption

The probability of resumption rose most steeply in the first 2 years of observation from age 20 years. When fitting the Cox regression model, there was evidence of violation of the proportional hazards assumption for some variables when examining resumption over the full follow-up period (see “Methods”). Table 4 therefore presents the Cox regression model for the 3 year period when cases were aged 20–22 years, where the proportional hazards assumption was met.

**Table 4:** Unadjusted and adjusted Cox regression model for resumption of prescription aged 20–22 years

Variable	Unadjusted		Adjusted	
	hazard ratio (95% CI)	*p* value	hazard ratio (95% CI)	*p* value
Female gender	1.91 (1.09–3.32)	0.02	1.81 (1.04–3.26)	0.05
Referral recorded to adult mental health services	3.27 (2.01–5.31)	< 0.0001	2.18 (1.30–3.66)	0.003
Learning disability	3.64 (1.98–6.70)	< 0.0001	2.43 (1.25–4.74)	0.009
Conduct disorder	1.32 (0.61–2.86)	0.48		
Autism spectrum disorder	1.99 (1.09–3.67)	0.03	1.47 (0.76–2.83)	0.26
Anxiety or depression	1.57 (0.83–2.96)	0.16		
Substance misuse	2.57 (1.04–6.33)	0.04	1.81 (0.72–4.56)	0.21
Antidepressant prescription aged 19 and over	2.08 (1.28–3.41)	0.004	1.08 (0.63–1.86)	0.77
Antipsychotic prescription aged 19 and over	6.93 (3.97–12.1)	< 0.0001	3.58 (1.86–6.88)	< 0.0001
Anxiolytic/hypnotic prescription aged 19 and over	3.20 (1.60–6.39)	< 0.001	1.83 (0.88–3.80)	0.10
Age at stopping medication (17 or 18 versus 14–16)	2.25 (1.43–3.53)	< 0.0001	2.04 (1.28–3.24)	0.003

In the adjusted model, female gender remained associated with an increased probability of resumption, as did a recorded referral to adult mental health services and having a recorded learning disability diagnosis. Prescription of antipsychotic medication was also strongly associated with resumption. Those that were older at the time of stopping medication also appeared to be more likely to restart within this time period.

### Comparison of non-ADHD psychotropic prescribing in individuals who did and did not resume ADHD prescriptions

As shown in Table 5, prescribing of all categories of non-ADHD psychotropic medication was more common in young people resuming prescriptions. Almost half of those who resumed ADHD prescriptions also had a primary care prescription for a non-ADHD psychotropic medication. Prescription of different categories of non-ADHD psychotropics (e.g., an antidepressant and an antipsychotic) was also more common in those with prescription resumption.

**Table 5:** Comparison of non-ADHD psychotropic prescribing in individuals who did and did not resume ADHD prescriptions

Prescription status	No resumption group (*N* = 1331)	Resumption group (*N* = 109)	*p* value for Chi squared test
Any non-ADHD psychotropic prescription, *n* (%)	343 (25.8%)	52 (47.7%)	< 0.0001
Antidepressant prescription, *n* (%)	228 (17.1%)	36 (33.0%)	< 0.0001
Antipsychotic prescription, *n* (%)	136 (10.2%)	22 (20.2%)	0.001
Anxiolytic/hypnotic prescription, *n* (%)	69 (5.2%)	13 (11.9%)	0.003
Multiple prescribing (more than one category of non-ADHD psychotropic), *n* (%)	79 (5.9%)	16 (14.7%)	< 0.0001

## Discussion

### Main findings

International studies have demonstrated that the majority of young people with ADHD stop medication by the age of 18 years [6–9]. Our findings indicate that only a small proportion subsequently resume their prescriptions in primary care. In our study, the younger the age at stopping medication, the less likely individuals were to resume in early adulthood. We found that those resuming prescriptions on average were diagnosed with more comorbid psychiatric conditions and were more likely to be prescribed multiple psychotropic medications, making their management more complex and reinforcing the importance of specialist psychiatric oversight [24].

### Discussion and comparison with existing literature

The low prevalence of resumption in our study is in line with earlier work by Wong et al. which examined prescribing between 1999 and 2006 [18]. Our more recent finding still demands discussion of the current context in which prescribing is taking place to consider the implications.

We found that the younger the age at stopping, the less likely cases were to resume their prescriptions. Continuing to take medication into late adolescence is logically likely to be a marker of severity of symptoms, although notably the age of stopping prescriptions was associated with resumption in our Cox regression even after adjusting for psychiatric comorbidities (often related to severity). It is possible that the young people stopping medication earlier in adolescence are those who experienced remission of their ADHD, and who consequently underwent a planned cessation of medication. Correspondingly, those who stopped later around the age of leaving child services may have encountered the ‘twilight zone’ of transition described by Young et al. [25], and the structural barriers to ongoing support and prescribing, including a lack of transition planning and protocols, and the well documented lack of services for adults with ADHD [15, 16, 26]. The ‘late stoppers’ could therefore represent a group more likely to have experienced a more sudden and unplanned cessation of their ADHD medication, and who might therefore seek to resume at a later point. Stopping early may be more likely among those who struggle to tolerate drug treatment or who derive little benefit—although the inclusion criteria for this study required cases to have a year or more of prescriptions, this does not equate to adherence to taking medication. Both processes are likely to have been in operation in the current study.

We found that female gender was also associated with an increased probability of resumption. It is feasible that gender differences in help-seeking behaviour exist which could influence re-engagement with services [27]; however, it has also been suggested that young women prescribed medication may be more impaired as they face higher barriers to diagnosis and treatment in the first place [28].

Overall, a higher proportion of those restarting medication had a psychiatric comorbidity recorded at some point, with the most common being anxiety and depression, but none of the comorbid conditions were significant predictors of resumption in our adjusted Cox model. It is worth noting that comorbid conditions recorded in primary care may also be mislabelled; Asherson et al. have argued that lack of recognition of the clinical presentation of ADHD in adulthood may lead to undertreatment, or to substitution of other psychotropic medication for ADHD medication if the symptoms are mistaken for the signs of other common psychiatric disorders [29].

A study of adults with a new diagnosis of ADHD in the CPRD noted high rates of primary care prescribing of non-ADHD psychotropic medication in this group, with 16% being prescribed antipsychotic medication [30]. In our analysis, those resuming prescription of ADHD medication were also more likely to be prescribed all types of psychotropic medication, which could represent an indicator of contact with specialist mental health services, particularly for those prescribed antipsychotics. We found that only a quarter of those resuming prescriptions actually had an adult mental health services referral coded in CPRD, suggesting that referrals may be under-recorded, and/or that some resumption may not be taking place under specialist supervision, possibly linked to a lack of appropriate services. This finding is similar to that reported by Bushe et al., who found that only a third of adults with an incident ADHD diagnosis had a secondary care referral recorded in CPRD [30].

### Strengths and limitations

This is the only study we are aware of to explore resumption of ADHD prescribing in early adulthood in UK primary care since the introduction of the 2008 NICE Guidance [19]. It uses the CPRD database which is broadly representative of the UK population and is likely to provide the fullest picture of prescribing due to widespread shared-care protocols for ADHD [20, 31]. Having said this, we acknowledge that our study is likely to represent an underestimate of resumption to some extent, for example in cases where ADHD medication is resumed and prescribed solely in specialist services, or by private psychiatrists.

The low proportion of cases with a recorded secondary care referral also suggests that referrals were under-recorded or perhaps recorded as free text (which is unavailable to researchers, for fear of de-anonymisation); this consideration does limit any conclusions we can draw about the level of oversight and specialist support for resumption [30].

This study examines only medication prescribing. There is no way of knowing whether or not the cases included in this study were actually taking their medication as prescribed, and whether they stopped medication when prescribing ceased or at a much earlier point. Non-adherence is estimated to be widespread in ADHD, and is also influenced by factors such as socioeconomic status, beliefs and attitudes and of course adverse effects and medication effectiveness, which we were unable to take into account when considering differences between those who did and did not resume their prescriptions [32, 33].

Unfortunately, our exploration of resumption in this respect was also limited by the absence of data on symptoms and severity of ADHD recorded in CPRD. Even so, we were able to examine age at stopping, recorded psychiatric comorbidities and non-ADHD psychotropic prescribing, all of which provide information characterising who did and did not resume prescriptions and can direct future research.

### Clinical implications

The low prevalence of prescription resumption in our study is perhaps surprising, given that we know that the rate of prescription cessation remains greater than the rate of symptom decline [1, 6–9]. The implication is that there is a population of young people who could benefit from resuming medication, but have not done so. Indeed, reports of structural barriers to prescribing in adults with ADHD mean that we cannot assume that all those who could benefit from resuming medication are able to do so [15, 24]. Qualitative research has also illuminated the journeys and struggles of adults seeking to re-enter services. In the recent CATCh-uS ADHD study, participants with ADHD who re-entered services in their 20s discussed their difficulties in managing their early adult lives without services or support [34]. Often, consequences such as job loss or criminal justice contact represented the triggers for them to attempt to re-enter services, a process which was sometimes prompted and assisted by a family member or partner. Both CATCh-uS and earlier work by Matheson et al. [35] highlighted the considerable persistence often required to seek and obtain support and treatment for ADHD as an adult. This suggests that many of the most vulnerable individuals with ongoing ADHD symptoms may not have the resources required to surmount the barriers to re-enter services.

Our research therefore adds support to the case for designing and commissioning more flexible models of prescribing support in ADHD, with easier exit and entry points. Brinkman et al.’s survey of participants in the MTA trial reported that many adolescents recognised the need for medication only after stopping, and suggested that a ‘start–stop–start’ pattern with the attendant flexibility may be their preferred model [12]. Early findings from the CATCh-uS study also illustrate how adolescents have differing conceptualisations of what ADHD means to them and what it means for their future as adults, which influence how ‘ready’ they are for transition and whether they ‘drift’ away from services [34]. Therefore, when and if adolescents stop their medication, acknowledging and discussing contingencies such as what to do if symptoms re-emerge or affect their adult life could be a helpful strategy.

However, alongside a need for flexibility, our findings also paint a picture of greater complexity in those who resume prescriptions, contributing to the debate about where ADHD is best managed for adults with the condition. As argued by Coghill, symptom monitoring, monitoring of impairment and functioning and monitoring of comorbid conditions are skilled and time consuming tasks which may be best suited to specialist care [24].

### Research implications

NICE guidance, and consensus statements outline recommendations on prescribing in ADHD, but research suggests implementation and service provision is highly variable [14, 15, 30, 34]. Very recent reports highlight concern that some UK Adult ADHD services may be in the process of being decommissioned, and others have expanding waits [36]. Our findings, in line with other research, also suggest that some adult ADHD prescribing may be taking place without specialist supervision, raising questions about how well GPs are being supported to take on sole responsibility for this aspect of care [14, 30]. Consequently, it is important to better understand the role of primary care, and the needs and perspectives of GPs; this is an area which has been little studied to date.

There is also a need to develop and evaluate models of managing ADHD in transition and early adulthood which address the tensions between prescribing for a potentially complex patient group, and a more flexible approach to medication management. In particular, findings from the MTA study and CATCh-uS suggest that the ‘stop–start’ pattern of medication is likely to be influenced by the patient’s current circumstances and context [12, 34]; future studies could test and explore flexible prescribing models further, considering patient preference, professionals perspectives’ and tracking outcomes [37]. It would also be helpful for future research using primary care databases to examine patterns of resumption beyond the age range of this study into the mid-thirties and beyond, as this would provide a fuller picture of adults seeking to re-engage with services and treatment.

## Conclusion

This study suggests that most of those who stop medication in adolescence do not have their prescriptions resumed in early adulthood. Those that could benefit from resuming their medication as adults need to be able to do so in a safe and stable manner in order to experience improved outcomes. Our findings raise questions around whether current models of care and prescribing are suitable and flexible enough for young adults with ADHD, and whether professionals in primary care are adequately supported by specialist services in managing this group. As a result, there is a need for studies to examine the barriers to implementation of guidance, and for evaluation to develop and refine feasible and acceptable models within an environment of resources constraints.
